# Efficient cellular annotation of histopathology slides with real-time AI augmentation

**DOI:** 10.1038/s41746-021-00534-0

**Published:** 2021-11-22

**Authors:** James A. Diao, Richard J. Chen, Joseph C. Kvedar

**Affiliations:** 1 Program in Health Sciences and Technology, Harvard Medical School, Boston, MA USA; 2 Department of Biomedical Informatics, Harvard Medical School, Boston, MA USA; 3 Department of Dermatology, Massachusetts General Hospital, Boston, MA USA

**Keywords:** Data acquisition, Image processing, Software

## Abstract

In recent years, a steady swell of biological image data has driven rapid progress in healthcare applications of computer vision and machine learning. To make sense of this data, scientists often rely on detailed annotations from domain experts for training artificial intelligence (AI) algorithms. The time-consuming and costly process of collecting annotations presents a sizable bottleneck for AI research and development. HALS (Human-Augmenting Labeling System) is a collaborative human-AI labeling workflow that uses an iterative “review-and-revise” model to improve the efficiency of this critical process in computational pathology.

Recent advances in computer vision and the rapid digitization of histology slides have escalated interest in artificial intelligence (AI) applications for pathology. Modern algorithms are not only capable of predicting regions of cancer,^1^ but also driver mutations,^2^ metastatic origins,^3^ and patient prognosis.^4^ Many algorithms rely only on the slide image and metadata, but others also require annotations of cells, tissues, and other entities within the slide. This additional information helps connect histology images to their underlying biology and may improve the interpretability and generalizability of resulting algorithms.^5^ Still, acquiring sufficient labels to train data-hungry models is a significant challenge, potentially requiring millions of annotations across thousands of slides.^5^ To address this bottleneck, van der Wal et al.^6^ introduce HALS (Human-Augmenting Labeling System) to enable efficient, high-quality annotations at scale.

HALS consists of three software components—a segmentation model, a classifier model, and an active learner model—that jointly support a human annotator. First, the segmentation model identifies the location of all cells in a small region. Second, the annotator, usually a board-certified pathologist, begins labeling cells. These labels are used to train a classifier model that identifies and labels other cells in the region that the annotator may correct. As the classifier learns from these corrections, its predictions progressively improve and require fewer corrections. When the first region is sufficiently labeled (20–30 annotations per class), the active learner model identifies the next most informative region for annotation, and the process repeats.

The advantages are two-fold. First, HALS’s classifier model reduces workload by replacing the laborious task of primary annotations with the simpler task of correction annotations. Second, HALS’s active learner model improves data efficiency by directing the annotator to more informative slide regions. To demonstrate these advantages, the authors measure workload (proportion of AI suggestions requiring correction) and data efficiency (validation accuracy vs. sample size) for seven pathologists using HALS to label tumor cells, tumor-infiltrating lymphocytes, eosinophils, and Ki-67^+^ cells. HALS resulted in significant workload reductions for all tasks, ranging from 96% for tumor cells (the easiest task) to 83% for eosinophils. In other words, a pathologist armed with HALS is expected to produce approximately 10 times more annotations with the same number of clicks. Algorithms trained using HALS were also 1.4–6.4% more data efficient than those trained without AI augmentation, suggesting higher quality labels. Improvements were robust across the four cell types and two stains, lending credence to the system’s generalizability. In addition to these objective improvements, AI augmentation also allows pathologists to monitor classifier improvement and identify failure modes throughout the labeling process. This may enable closer collaboration with engineers and contribute insights for understanding model deficiencies and allocating labeling resources.

Similar “review-and-revise” systems are widely commercialized and have been utilized in areas such as object recognition,^7^ autonomous driving research,^8^ and dermatologic classification.^9^ HALS offers several unique contributions for pathology applications. The active learning component^10^ is particularly well-suited for digital pathology, where gigapixel-resolution images preclude exhaustive annotation. In addition, HALS synthesizes several pathology-specific segmentation,^11^ classification,^12^ and interface^13^ systems into a single modular framework that simplifies application and adaptation. Future work should investigate scalability to large slide repositories, region selection heuristics that prioritize cell classes in greatest need of improvement, and application areas beyond microscopy.

In past decades, the decreasing cost of sequencing has powered explosive progress in genetics.^14^ Overcoming the high cost of expert annotations may unlock similar possibilities for the continuing deluge of biological image data. In their article, Van der Wal and colleagues present compelling results that labeling augmentation with HALS may decrease annotation cost up to 10-fold, indicating substantial progress toward this goal. For academic investigators, AI augmented labels could enable more rapid and granular study of biological hypotheses. For industry teams, newly freed resources and higher quality data may accelerate development of AI-based software-as-a-medical-device (SaMD). As systems like HALS continue to improve, we anticipate that AI augmentation may soon become an ubiquitous aid for embedding human knowledge into data.
